# Combined inhibition of complement and CD14 improved outcome in porcine polymicrobial sepsis

**DOI:** 10.1186/s13054-015-1129-9

**Published:** 2015-11-27

**Authors:** Espen W. Skjeflo, Caroline Sagatun, Knut Dybwik, Sturla Aam, Sven H. Urving, Miles A. Nunn, Hilde Fure, Corinna Lau, Ole-Lars Brekke, Markus Huber-Lang, Terje Espevik, Andreas Barratt-Due, Erik W. Nielsen, Tom E. Mollnes

**Affiliations:** Research Laboratory, Nordland Hospital, Prinsens Gate 164, 8092 Bodø, Norway; Faculty of Health Sciences, K. G. Jebsen TREC, University of Tromsø, 9037 Tromsø, Norway; Department of Surgery, Nordland Hospital, Prinsens Gate 164, 8092 Bodø, Norway; Department of Anestesiology, Nordland Hospital, Prinsens Gate 164, 8092 Bodø, Norway; Faculty of Professional Studies, University of Nordland, Universitetsaleen 11, 8049 Bodø, Norway; Faculty of Medicine, Ludwig Maximillian University, Professor Huber Platz 2, 80539 Munich, Germany; Volution Immuno Pharmaceuticals Limited, 5 Argosy Court, Whitley Business Park, Coventry, CV3 4GA UK; Department of Traumatology, Center of Surgery, University of Ulm, Albert Einstein Allee 23, 89081 Ulm, Germany; Centre of Molecular Inflammation Research, and Department of Cancer Research and Molecular Medicine, Norwegian University of Science and Technology, Høgskoleringen 1, 7491 Trondheim, Norway; Department of Immunology, Oslo University Hospital and K.G. Jebsen IRC, University of Oslo, PB 4960 Nydalen, 0424 Oslo, Norway; Division of Emergencies and Critical Care, Rikshospitalet, Oslo University Hospital Oslo, Sognsvannsveien 20, 0372 Oslo, Norway

## Abstract

**Introduction:**

Sepsis is an exaggerated and dysfunctional immune response to infection. Activation of innate immunity recognition systems including complement and the Toll-like receptor family initiate this disproportionate inflammatory response. The aim of this study was to explore the effect of combined inhibition of the complement component C5 and the Toll-like receptor co-factor CD14 on survival, hemodynamic parameters and systemic inflammation including complement activation in a clinically relevant porcine model of polymicrobial sepsis.

**Methods:**

Norwegian landrace piglets (4 ± 0.5 kg) were blindly randomized to a treatment group (n = 12) receiving the C5 inhibitor coversin (OmCI) and anti-CD14 or to a positive control group (n = 12) receiving saline. Under anesthesia, sepsis was induced by a 2 cm cecal incision and the piglets were monitored in standard intensive care for 8 hours. Three sham piglets had a laparotomy without cecal incision or treatment. Complement activation was measured as sC5b-9 using enzyme immunoassay. Cytokines were measured with multiplex technology.

**Results:**

Combined C5 and CD14 inhibition significantly improved survival (*p* = 0.03). Nine piglets survived in the treatment group and four in the control group. The treatment group had significantly lower pulmonary artery pressure (*p* = 0.04) and ratio of pulmonary artery pressure to systemic artery pressure (*p* < 0.001). Plasma sC5b-9 levels were significantly lower in the treatment group (*p* < 0.001) and correlated significantly with mortality (*p* = 0.006). IL-8 and IL-10 were significantly (*p* < 0.05) lower in the treatment group.

**Conclusions:**

Combined inhibition of C5 and CD14 significantly improved survival, hemodynamic parameters and inflammation in a blinded, randomized trial of porcine polymicrobial sepsis.

## Introduction

Sepsis presents with the cardinal signs of inflammation on the basis of a suspected or detected infective agent [1]. If left untreated, or treated too late, sepsis will progress to a systemically uncontrolled, dysregulated immune response leading to vascular instability, capillary leakage, septic cardiomyopathy, shock, disseminated intravascular coagulation, and acute kidney and respiratory failure. This multiple organ failure (MOF) is associated with high costs of care, and more importantly high rates of morbidity and mortality [2, 3].

Despite much research and several attempts to provide specific drug therapies the treatment is still early application of broad-spectrum antibiotics and supportive therapy [4]. With the withdrawal of Xigris (drotrecogin alpha) in 2011, more effective and specific treatment strategies of sepsis are needed [5]. There is also a need for adequate experimental models of sepsis as the most commonly used animal models have failed to mimic human sepsis [6, 7].

Early studies focused on the endotoxic shock caused by intravenous (IV) infusion of lipopolysaccharide (LPS), bacterial infusions or cecal ligation and puncture (CLP) in smaller animals such as mice and rats. Now more elaborate, sophisticated, and clinically relevant models in larger animals are emerging [8]. In experimental sepsis the model of cecal incision in pig overcomes several pitfalls. Compared to rodents, pigs have a greater genetic, anatomical and physiological resemblance to humans [9–11]. Furthermore, their size enables repeated blood sampling for comprehensive analysis and the use of human medical equipment. Finally, exposing the pig to its own microbiota by cecal incision induces polymicrobial sepsis with a defined site of infection, often lacking in other experimental models. The model presented here is very close to clinical conditions like intestinal leak during anastomotic insufficiency, bowel perforation or bacterial translocation following conditions of sterile inflammation in the abdomen.

As the first line of defense, innate immunity acts upon infection with activation through pattern recognition. In sepsis, this activation is exaggerated and worsens the disease through overproduction of multiple cytokines, paracrine and autocrine factors. In numerous crisscrossing pathways the inflammatory network acts on the vasculature and organs [10, 12]. In the early phase, the Toll-like receptor (TLR) and complement systems together constitute the primary pathways of danger recognition through pattern recognition receptors (PRRs) and consequently, immune activation [13, 14]. The three pathways of complement activation converge on component C3, which proceeds to activate complement component 5 (C5), generating C5a and the final, terminal C5b-9 complement complex (TCC) and C5a has a documented, harmful role in sepsis [15]. Thus, C5 is a particularly interesting target for intervention in sepsis, leaving C3 free to opsonize in microbial defense. Equally, cluster of differentiation (CD)14 is an important coreceptor to several of the TLRs, including TLR4, TLR2, TLR3, TLR7 and TLR9 [16].

We have therefore proposed inhibition of TLR and complement as a treatment regimen to attenuate the septic inflammatory response [17]. Recently, we showed convincing data in a murine CLP model of sepsis that supports this approach [18]. The aim of the current study was to test our hypothesis by inhibiting both complement and TLR. We specifically inhibited the key proinflammatory components C5 and CD14 in a blinded, randomized, prospective study of porcine polymicrobial sepsis that closely mimics clinical sepsis.

## Methods

### Treatment reagents

Coversin, the 16.8 kDa, endotoxin-free recombinant bacterial *Ornithodoros moubata* complement inhibitor (OmCI; coversin) protein [19], was kindly provided by Volution Immuno Pharmaceuticals Ltd (Coventry, UK). The mouse anti-porcine CD14 IgG2/4 chimeric monoclonal antibody clone rMIL-2 has been described in detail previously [20] and was produced by Excellgene SA (Monthey, Switzerland).

### Animals, anesthesia and monitoring

Norwegian landrace piglets (*Sus scrofa domesticus*) (n = 24), weighing 4 ± 0.5 kg were obtained from an authorized industrial pig producer. Approximately 2 mg/kg intranasal midazolam was administered before gaining intravascular access to an ear vein (2.5 mg/mL or 5 mg/mL). Piglets then received 10–15 mg/kg ketamine prior to endotracheal intubation with a 4.0 mm inner diameter tube with cuff. After intubation, IV infusions of propofol (10 mg/kg/hr) and fentanyl (50 μg/kg/hr) were initiated. Four piglets received small IV boluses of pancuronium bromide when shivering interfered with ventilation. Mechanical ventilation was provided and the following volume-controlled mode was used: tidal volume of 15–20 mL/kg, respiratory rate of 26 per minute, inspiratory/expiratory time ratio of 1:3, positive end-expiratory pressure of 0 cm H_2_O, and inspiratory oxygen fraction of 0.3. The respiratory settings were adjusted to maintain pH at 7.4 only before the surgical cecal incision. All subsequent changes in arterial blood gas values were considered a result of sepsis, and the respiratory settings were left unchanged. An arterial catheter was surgically inserted into the internal carotid artery and a 5-French Edwards Swan-Ganz catheter (Baxter Healthcare Corp., Irvine, CA, USA) was inserted in the external jugular vein and advanced until a satisfactory pulmonary artery occlusion pressure was obtained. All pressure transducers were zero-referenced to midchest level. With a minimally invasive technique, a urinary catheter was inserted during the laparotomy to monitor urinary output and core temperature. Temperature, heart rate (HR), mean systemic arterial pressure (MAP), mean pulmonary artery pressure (MPAP), central venous pressure and pulmonary artery occlusion pressure were displayed continuously on a Siemens SC 8000 Patient Monitor (Healthcare Diagnostics Ltd., Camberley, UK) and values recorded every 5 minutes.

### Animal model and experimental design

Piglets were allocated to receive either treatment (n = 12) or saline as vehicle (n = 12) before surgical sepsis was induced. Each week, two piglets were randomized as matched pairs to maintain homogeneity in regard to weight, age and gender, yielding 12 pairs of piglets receiving treatment or serving as the untreated counterpart. All investigators participating in the experiment were blinded to this allocation. Finally, three piglets served as sham controls, having a laparotomy and cystotomy but not a cecal incision. After baseline blood samples were obtained, the interventional reagents were then administered as follows: in the treatment group, an initial IV infusion of coversin (1 mg/kg) was delivered as a 5-minute bolus followed by a second 5-minute bolus containing the mouse anti-porcine CD14 monoclonal antibody (5 mg/kg). Immediately after the anti-CD14 bolus, a continuous IV infusion of coversin was initiated at 0.05 mg/kg/h and maintained throughout the experiment. For the untreated group, saline was administered in the exact same manner regarding volume and infusion time.

Piglets then had a laparotomy with a 10 cm longitudinal incision in the right lower lumbar region, the cecum was localized and a 2 cm longitudinal incision made. Intestinal content was allowed to leak into the abdominal cavity before closing the abdomen in two layers, as instructed by Kato and colleagues [8]. Piglets were then observed until death or otherwise for 8 hours at which point they were euthanized by propofol (50 mg), fentanyl (200 μg), and KCl (10 mmol).

### Blood samples

Blood samples were drawn at baseline (immediately after IV access and before surgery) immediately after surgery and 1.5, 3, 4, 5, 6 and 8 hours after surgery. From those animals that died before 8 hours, a final sample was drawn at death. Whole blood was used for arterial blood gas analysis, performed on an Epoc reader (Epocal, Inc., Ottawa, ON, Canada). Hematological parameters including hemoglobin, hematocrit, total leukocyte and platelet counts were analyzed on a Siemens ADVIA 2120 instrument (Siemens Healthcare Diagnostics Ltd., Camberley, UK). Serum was obtained after clotting in gel tubes from Greiner Bio-One GmbH (Frickenhausen, Germany). Serum creatinine, alanine transaminase (ALT), aspartate aminotransferase (AST), creatine kinase (CK), albumin and total protein were analyzed using ADVIA®1800 (Siemens Medical Solutions Diagnostics, Japan) using reagents from Siemens Healthcare Diagnostics Ltd. (Camberley, UK).

### Blood bacterial culture

Samples for detection of bacteria in blood were obtained at baseline (before surgery) and at death using routine sampling and assays according to the procedures at the hospital's intensive care unit.

### Complement activation and cytokines

EDTA-plasma was prepared by immediately placing the blood sample on crushed ice. Within 30 minutes samples were centrifuged at 2500 × *g* for 15 minutes at 4 °C and plasma immediately stored at −70 °C. Complement activation was measured as the soluble TCC (sC5b-9) using multiplex xMAP technology (Bio-Plex; Bio-Rad Laboratories, Inc., Hercules, CA, USA) as previously described [21]. Briefly, mouse anti C5b-9 Ab (clone aE11; Diatec Monoclonals AS, Oslo, Norway) was coupled to carboxylated magnetic beads (Bio-Plex Pro) using a Bio-Plex amine coupling kit (Bio-Rad Laboratories, Inc.). Coupled beads were then incubated with samples, followed by biotinylated anti-C6 monoclonal antibody (mAb) (Quidel, San Diego, CA, USA) and streptavidin-PE (Bio-Rad Laboratories, Inc.). The international complement standard number 2, recently described [22], was used as the calibration curve. Measurement and data analysis was performed with the Bio-Plex™ 200 system and the Bio-Plex Manager software version 6.0. Porcine cytokines (n = 9) were analyzed in plasma using a multiplex kit from Bio-Rad Laboratories, Inc. according to the manufacturer’s instructions. The kit comprised the following cytokines: interferon (IFN)-α, IFN-β, tumor necrosis factor (TNF), interleukin (IL)-1β, IL-4, IL-6, IL-8, IL-10 and IL-12p40.

### Ethics

The animals were treated in accordance with the Norwegian Laboratory Animal Regulations and the EU directive 2010/64/EU. The Norwegian Animal Research Authority approved the study.

### Statistics

All statistics were performed using GraphPad Prism 6 for Mac (GraphPad Software, San Diego, CA, USA). Serial measurements were analyzed by comparing descriptive summary measures (means and differences between maximum and minimum values) as described for peaked and growth data [23]. Survival curves were compared using log-rank (Mantel-Cox) test and correlation was determined by calculating the Pearson coefficient. The treated group was compared to the untreated group using the nonparametric Wilcoxon matched-pairs signed-rank test according to the matched-pairs design of the study. *p* < 0.05 was considered significant.

## Results

### Effect of treatment on survival during polymicrobial sepsis

**Fig. 1:**
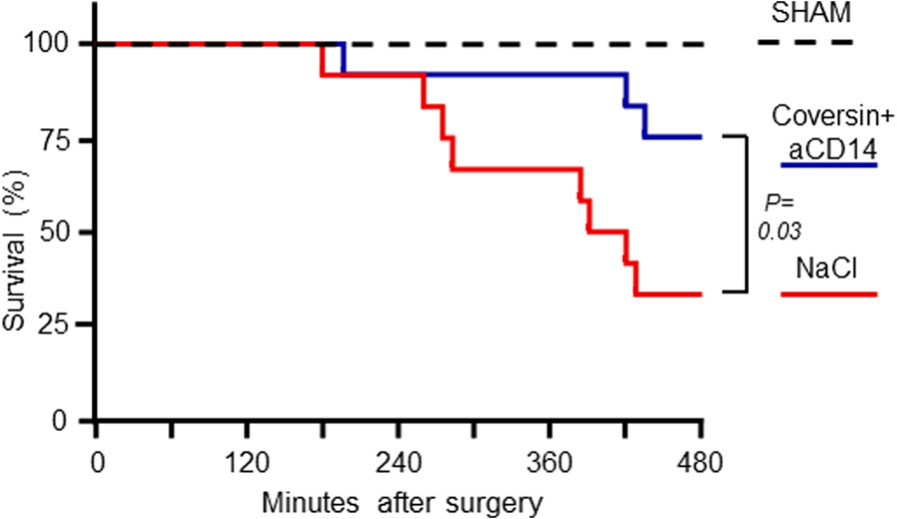
Effect of treatment with C5 and CD14 inhibitors on survival in porcine polymicrobial sepsis. Piglets were randomized to receive coversin (C5 inhibitor) and anti-CD14 as treatment (n = 12) or saline (n = 12) in a cecal incision model of sepsis. Sham animals (n = 3) did not undergo cecal incision and were included for reference. Mortality was recorded during the 8-hour period and plotted as a Kaplan-Meier curve. The curves were analyzed using the log-rank (Mantel-Cox) test. *C5* complement component 5, *CD* cluster of differentiation

A significantly reduced mortality was observed in the treated group where nine out of 12 animals survived during the 8-hour observation period, compared to four out of 12 in the nontreated group (*p* = 0.03) (Fig. 1). All three uninfected (sham) animals survived throughout the 8-hour observation time.

### Effect of treatment on mean systemic and pulmonary arterial pressure

**Fig. 2:**
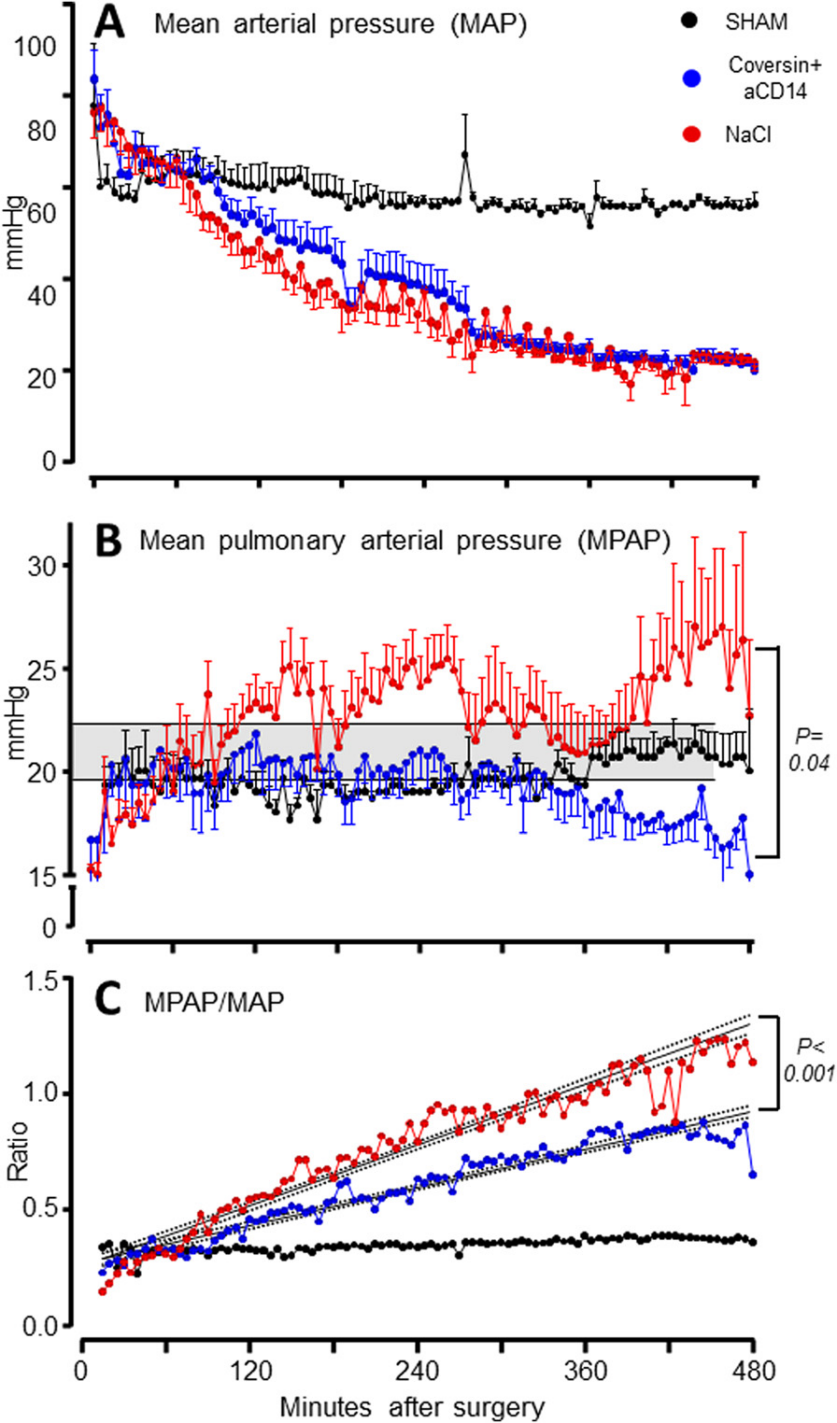
Effect of treatment with C5 and CD14 inhibitors on hemodynamics in porcine polymicrobial sepsis. Mean systemic arterial pressure (MAP) (panel **a**) and mean pulmonary artery pressures (MPAP) (panel **b**) for each subject were recorded every 5 minutes. The plots represent mean values with corresponding standard errors of means. The shaded area in panel **b** represents the difference in mean MPAP whereas the mean ratio of the MPAP to MAP is plotted in (panel **c**). Lines are drawn by simple linear regression. The sham animal curve is included for reference. *C5* complement component 5, *CD* cluster of differentiation

Hemodynamic parameters were analyzed in search of an explanation for the improved survival. Following surgery, all animals with cecal perforation had a higher heart rate (HR) (mean 183 bpm) compared to the animals in the sham group (mean 134 bpm). The HR peaked 2 hours after perforation. Following a brief increase in mean arterial pressure (MAP), both the treatment and the control groups developed classical signs of septic shock with a decrease in MAP (Fig. 2a). The mean pulmonary arterial pressure (MPAP) increased substantially in the nontreated group during the course (Fig. 2b). In comparison, this increase was not shown in the treated group (*p* = 0.04) (Fig. 2b). The MPAP/MAP ratio, reflecting the changes in the normally low-pressure pulmonary circuit compared to the normally high-pressure systemic circuit, increased in both the treated and the untreated group (Fig. 2c). Notably, the treated group had a significantly lower mean MPAP/MAP ratio than the untreated group (*p* = 0.016), and the ratio increased significantly less than in the untreated group when the trajectory of increase was compared by linear regression (*p* < 0.001) (Fig. 2c).

### Effect of complement activation on treatment and survival

**Fig. 3:**
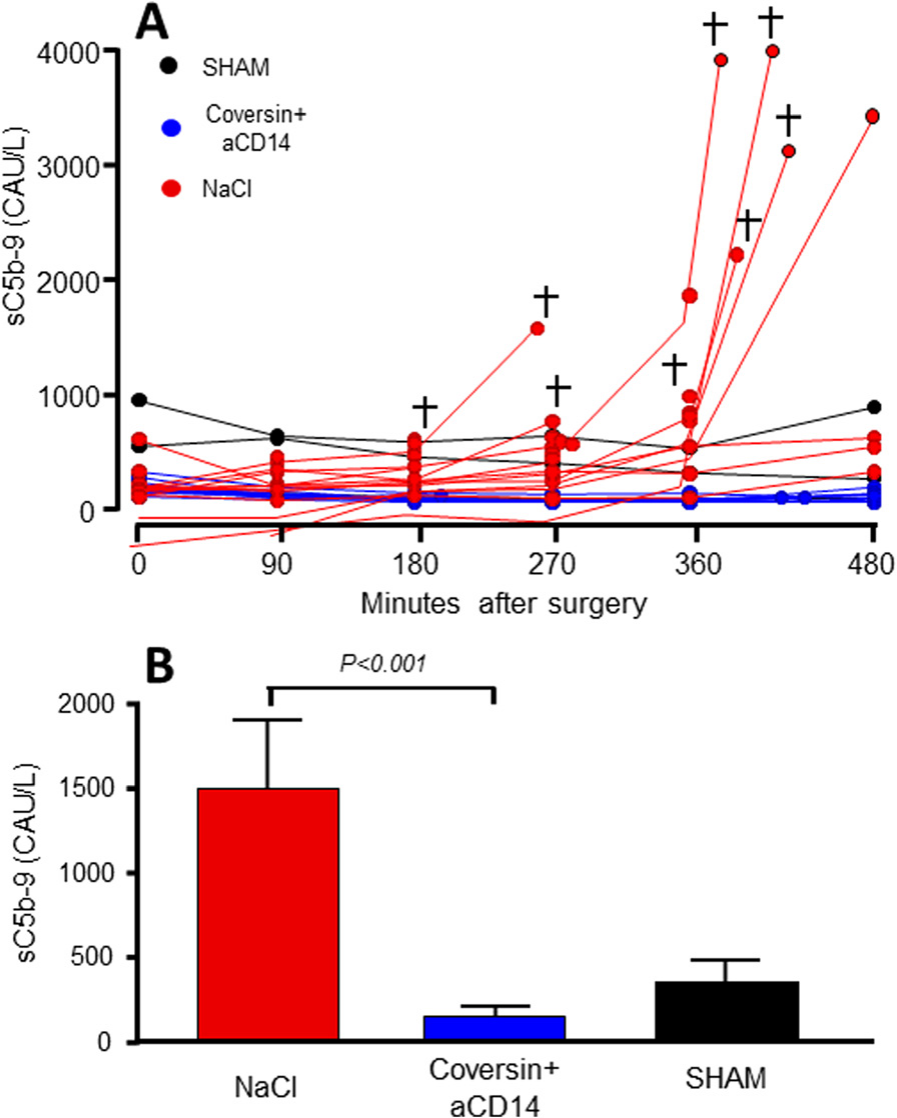
Effect of treatment with C5 and CD14 inhibitors on complement activation in porcine polymicrobial sepsis. The plasma concentration of sC5b-9 was determined every 90 minutes during the 8-hour observation and additional samples were obtained from animals that died before the end of the observation period (panel **a**). The difference in mean increase from minimum to maximum values of complement activation between the treated and control groups are shown in (panel **b**). The sham animal data is included for reference. † = death before the end of the observation period. CAU = complement arbitrary units, see ref. [22]. *C5* complement component 5, *CD* cluster of differentiation, *sC5b-9* soluble terminal C5b-9 complement complex

Seven of the eight animals in the untreated group that died before scheduled euthanasia had exponentially increased sC5b-9 levels (range 444–3713 complement arbitrary units (CAU)/L). (Fig. 3a). sC5b-9 levels were significantly higher in the untreated group compared to treated animals (*p* < 0.001, Fig. 3b). In the treated group, all but one animal had a net decrease in sC5b-9 levels to below baseline values throughout the experiment, consistent with a complete blockade of C5 and thus no substrate for sC5b-9 generation. We then compared the levels of sC5b-9 to the outcome for all 27 animals in a 2 × 2 plot using Fisher's exact test for contingency, and found a significant association between sC5b-9 levels and death (*p* = 0.006; odds ratio (OR) 25.00; 95 % confidence interval (CI) 2.094 to 298.5).

### Effect of complement and CD14 inhibition on inflammation, hematology and clinical-chemical parameters

**Fig. 4:**
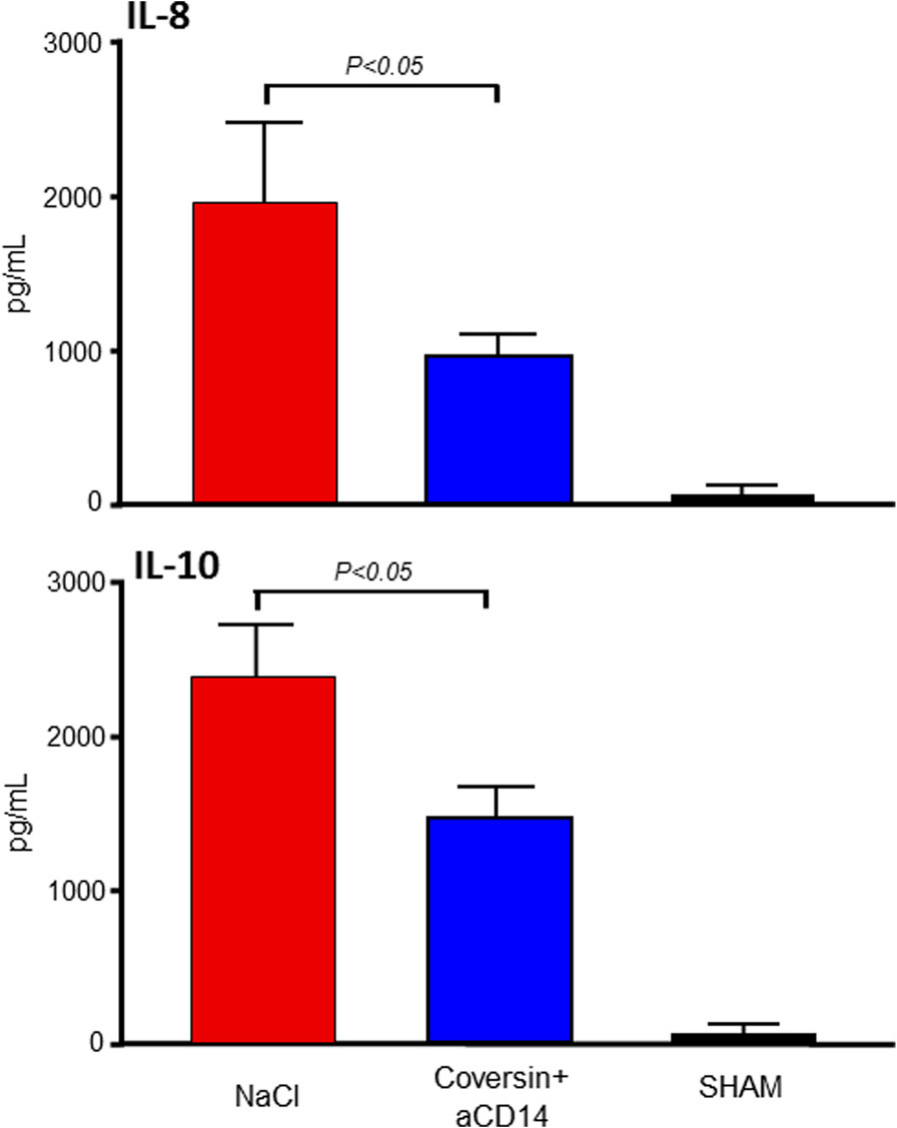
Effect of treatment with C5 and CD14 inhibitors on IL-8 and IL-10 in porcine polymicrobial sepsis. Plasma cytokines were measured during the observation period. The difference in mean increase from minimum to maximum values of plasma IL-8 and IL-10 are shown. The sham animal data is included for reference. *C5* complement component 5, *CD* cluster of differentiation, *IL* interleukin

The levels of IL-6, IL-8, IL-10, TNF and IL-12p40 increased in the untreated animals. A significant reduction of IL-8 (51 %) and IL-10 (39 %) (*p* < 0.05 for both) was observed in the treatment group compared to the untreated group (Fig. 4). Hematological and clinical-chemical parameters were largely similar in the two groups, except for a significantly lower mean total protein level in the treated group (Table 1).

**Table 1:** Comparison of hematological and clinical-chemical parameters in treated (coversin + anti-CD14) and untreated (Nacl) groups of piglets with polymicrobial sepsis

	NaCl	Coversin + anti-CD14	Untreated vs. treated	
	Change^a^ (Mean^c^)	Change (Mean)	Difference (Mean diff.)	P-value^b^ (P-value^d^)
Hemoglobin (g/dL)	2.2 ± 0.21 (10)	2.2 ± 0.28 (10)	0.0 ± 0.35 (0)	0.87 (0.42)
Leukocytes (10^9^/L)	5.7 ± 0.89 (5.8)	5.8 ± 0.78 (5.9)	0.1 ± 1.38 (0.1)	>0.99 (>0.99)
Thrombocytes (10^9^/L)	205 ± 37 (296)	198 ± 33 (230)	7 ± 37 (66)	0.78 (0.30)
Creatinine (μmol/L)	64 ± 8.9 (79)	73 ± 6.5 (88)	9 ± 7.2 (9)	0.15 (0.18)
ALAT (U/L)	18 ± 8.2 (43)	14 ± 2.3 (39)	4 ± 9.3 (4)	0.41 (0.91)
ASAT (U/L)	99 ± 21 (54)	94 ± 16 (52)	5 ± 30 (2)	0.81 (0.85)
Creatinine kinase (U/L)	305 ± 57 (448)	434 ± 69 (513)	129 ± 63 (65)	0.13 (0.30)
Albumin (g/L)	8.1 ± 0.6 (24)	10 ± 0.8 (23)	1.9 ± 1.2 (1)	0.20 (0.18)
Total protein (g/L)	12 ± 1.1 (37)	15 ± 1.5 (34)	3 ± 2.0 (3)	0.27 (0.02*)

^a^Change represents the difference between maximum and minimum values ± SEM

^b^The difference in change in the two groups was compared using Wilcoxon matched-pairs signed rank test

^c^Mean level throughout the experiment

^d^The difference in means (mean diff.) in the two groups was also compared using the Wilcoxon matched-pairs signed rank test. * P < 0.05 was considered significant

### Bacteriology

Polymicrobial bacteremia was confirmed in selected blood cultures revealing *Escherichia coli* and enterococci in all samples. Varying amounts of *Staphylococcus aureus*, streptococci and *Klebsiella* were also detected (data not shown).

## Discussion

The combined regimen of coversin and anti-CD14 was recently proven effective in reducing the mortality in a mouse CLP model [18]. However, in the present porcine study, the transfer of these experiments to a surgically relevant, human-like acute and severe septic state revealed an improved survival in the treated group that was greater than anticipated. Although all animals developed severe tachycardia and prolonged hypotension, a remarkable difference between the two groups was observed. These piglets were of the same age, gender and farrow, selected at the same time of year with equal diets and conditions before the experiment. Furthermore, the animals were randomly selected as matched pairs, the intervention was unknown to the investigators and the animals received no other form of intervention or supportive therapy than that described.

Our analysis revealed subtle but distinct differences in MAP throughout the experiment but the greatest effect was seen on MPAP and MPAP in relation to MAP. On average, both groups had a lower MAP throughout the experiment, but the untreated group showed a more rapid decrease in MAP than the treated group. Clinically, both groups developed septic shock. However, the untreated group had a significantly greater increase in MPAP reaching higher maximum values than the treated group. Indeed, the MPAP in the treated group was comparable to sham for a long time period of the experiment. Notably, as shown by Kato and colleagues [8], the MPAP to MAP ratio can be calculated to reveal changes in both systemic and pulmonary circulation in sepsis; as the systemic pressure falls and the pulmonary pressure increases, the two merge as the factor approaches 1.0. In our study, the dynamic increase of MPAP in relation to decrease in MAP was significantly less pronounced in the treated group, underscoring a beneficial effect of blocking C5 and CD14 on the hemodynamic parameters.

In contrast to humans, pigs harbor large amounts of pulmonary intravascular macrophages serving as the predominant part of the mononuclear phagocytic system [24]. There are several reports on porcine pulmonary hypertension following infusion of endotoxin [25, 26] triggering the release of prostaglandins, leukotrienes and other vasoactive mediators from macrophages. In this study, we can speculate that the combined treatment abolished recognition of endotoxin and other pathogen-associated molecular patterns, thus reducing the ensuing inflammation and vasoconstriction. The described leukotriene B4 (LTB4) capture by coversin could also enhance this effect.

Coversin has an internal binding pocket capturing LTB4 [27] and thus has the potential to reduce LTB4-dependent effects in sepsis. In turn, LTB4 promotes neutrophil chemotaxis, increases adherence to capillary walls and may directly induce pulmonary arterial hypertension [28]. In sepsis, neutrophil-mediated tissue injury often affects remote organs, particularly the lungs. In this context, impaired lung function often correlates with the intensity of neutrophilic infiltrates [29]. As coversin’s C5 and LTB4 binding activities are considered independent, producing a type of coversin that inhibits C5 but is unable to bind LTB4 could be used to further explore the selective contribution of LTB4 in this model [27].

Complement activation was markedly increased in the untreated group whereas it was nonexistent or even decreased in the treated group, consistent with a complete block of C5 and thus no substrate for sC5b-9 formation. Excessive systemic complement activation with liberation of C5a has a number of concomitant detrimental effects both directly and through other inflammatory and hemostatic mediators [30]. Thus, complement-mediated endothelial cell activation, which was not measured in the present study, could likely have contributed to prothrombotic activity leading to intravascular coagulation. This would eventually reduce tissue perfusion and contribute to the hemodynamic instability subsequently leading to organ failure. Within the short observation time, all animals that died in the untreated group had substantially elevated sC5b-9 levels, consistently escalating before death. In return, three of the four animals that survived in the untreated group did not show this increase. Arguably, the anticipated lack of activation could have protected the treated group by lowering ischemia-induced inflammation during shock. In this line of thought, complement activation can be considered as a cause of increased morbidity and mortality in the untreated group. As a limitation of the study, a direct causal effect of complement activation alone cannot be proven from the present data. However, the increase in the activation product sC5b-9 seems to be a good marker for the processes affecting outcome. A similar close and highly significant correlation has been described between the levels of plasma sC5-9 in patients admitted to hospital with *Neisseria meningitidis* sepsis and the mortality during the course of this disease [31].

Downstream effects of complement and TLR activation include the release of cytokines and chemokines. We therefore examined whether inhibition of complement and CD14 would have any effect on these inflammatory mediators. We documented a modest but distinct increase in several cytokines during the course of sepsis. Importantly, for IL-8 and IL-10, this increase was significantly less pronounced for the treated group, indicating an early effect of the treatment regimen on these two key cytokines. Increased levels of both the proinflammatory chemokine IL-8 and the anti-inflammatory cytokine IL-10 have been associated with severe sepsis and poor outcome [32–34]. In models of both Gram-negative and Gram-positive bacteria-induced inflammation they are both highly dependent on activation of complement and CD14, IL-8 being relatively more complement-dependent [35, 36]. This is in accordance with our present findings in polymicrobial sepsis where complement activation was completely abolished in the treated group, which probably is a main explanation for the reduced IL-8 release, although an additional effect of blocking CD14 is likely for both mediators. In order to attenuate the proinflammatory cytokine storm as well as the anti-inflammatory immunosuppressive state, we claim that early reduction of IL-8 and IL-10 might be beneficial in sepsis.

In the present study, however, the cytokine response was generally less pronounced than expected from such an aggressive model. This suggests that the rapid and extensive complement activation per se may play a major role at this early stage where the full-fledged systemic inflammatory response syndrome (SIRS) and associated MOF did not have time to fully develop. Accordingly, few differences were seen in the hematological and clinical-chemical parameters reflecting organ damage. Yet, the unique finding of reduced mean total protein in the untreated group could be an early sign of capillary leak and therefore endothelial barrier dysfunction, which reportedly is one of the most important pathophysiological mechanisms in sepsis [37]. We do admit that the relatively short observational period as well as demanding nonparametric statistical analysis with increased risk of type II errors should be taken into consideration when interpreting the data.

Lastly, the blood cultures revealed growth of both Gram-negative and Gram-positive bacteria, indicating a clinically relevant model of polymicrobial sepsis. We have previously shown that combined C5 and CD14 inhibition efficiently inhibits inflammation induced by both Gram-negative [38] and Gram-positive [36] bacteria. Furthermore, we have shown that this combined inhibition, but not single inhibition of C5 and CD14, increased survival in a mouse CLP model [18]. In the present study we document for the first time that the combined regimen works in a large animal model of sepsis. It resembles human disease more closely and is clinically most relevant. The limited amount of inhibitors available and their high costs precluded us, however, from using adult pigs. Thus, the use of small piglets makes this model more relevant for neonatal sepsis than for adult sepsis, which also should be taken into consideration when interpreting the data. Finally, the fact that treatment was started at baseline, according with the “proof-of principle” of the protocol, is a limitation of the study with respect to clinical relevance for established sepsis. Recent data obtained in vitro using the combined complement- and CD14-inhibitory regimen “post challenge” indicates that a “therapeutic window” might exist [39], though an efficient effect of delayed treatment in vivo remains to be shown.

## Conclusions

From the results of the present study it is tempting to speculate that the combined inhibition of C5 and CD14 reduced the innate immune response to the extracompartmental gastrointestinal flora of the piglets, in turn reducing the derangement of the pulmonary circulation and thereby improving survival in this robust 8-hour model of sepsis. The need for antibiotic therapy in a clinical situation is obvious. Our hypothesis merits further testing in larger, longer and more comprehensive trials where the combined regimen we describe here is used to supplement conventional therapy.

## Key messages

- Treatment of sepsis is largely limited to antibiotics and supportive therapy. New specific treatment strategies are demanded as well as experimental models relevant for human sepsis.
- Systemic inhibition of two innate immune recognition systems was tested as a therapeutic strategy in a clinically relevant porcine model of sepsis.
- Combined inhibition of the complement component C5 and the TLR coreceptor CD14 abrogated complement activation, attenuated physiological derangements, reduced cytokine release and improved survival in a porcine model of polymicrobial sepsis.
- This broad-acting “upstream” inhibition of innate immunity might be a therapeutic approach to human sepsis, where inhibition of “downstream” single inflammatory mediators has failed.

## Abbreviations

C5: Complement component 5
CAU: Complement arbitrary units
CD: Cluster of differentiation
CLP: Cecal ligation and puncture
HR: Heart rate
IFN: Interferon
IL: Interleukin
IV: Intravenous
LPS: Lipopolysaccharide
LTB4: Leukotriene B4
MAP: Mean arterial pressure
MOF: Multiple organ failure
MPAP: Mean pulmonary artery pressure
OmCI: Ornithodoros moubata complement inhibitor
PRR: Pattern recognition receptor
sC5b-9: Soluble TCC
TCC: Terminal C5b-9 complement complex
TLR: Toll-like receptor
TNF: Tumor necrosis factor
